# Auditory, tactile, and multimodal noise reduce balance variability

**DOI:** 10.1007/s00221-023-06598-6

**Published:** 2023-03-24

**Authors:** Sam Carey, Jessica M. Ross, Ramesh Balasubramaniam

**Affiliations:** 1 Sensorimotor Neuroscience Laboratory, Cognitive & Information Sciences, University of California, 5200 N Lake Road, Merced, CA 95343 USA; 2 Veterans Affairs Palo Alto Healthcare System and the Sierra Pacific Mental Illness, Research, Education, and Clinical Center, Palo Alto, CA USA; 3 Department of Psychiatry and Behavioral Sciences, Stanford University Medical Center, Stanford, CA USA

**Keywords:** Postural sway, White noise, Auditory feedback, Tactile feedback

## Abstract

Auditory and somatosensory white noise can stabilize standing balance. However, the differential effects of auditory and tactile noise stimulation on balance are unknown. Prior work on unimodal noise stimulation showed gains in balance with white noise through the auditory and tactile modalities separately. The current study aims to examine whether multimodal noise elicits similar responses to unimodal noise. We recorded the postural sway of healthy young adults who were presented with continuous white noise through the auditory or tactile modalities and through a combination of both (multimodal condition) using a wearable device. Our results replicate previous work that showed that auditory or tactile noise reduces sway variability with and without vision. Additionally, we show that multimodal noise also reduces the variability of sway. Analysis of different frequency bands of sway is typically used to separate open-loop exploratory (< 0.3 Hz) and feedback-driven (> 0.3 Hz) sway. We performed this analysis and showed that unimodal and multimodal white noise affected postural sway variability similarly in both timescales. These results support that the sensory noise effects on balance are robust across unimodal and multimodal conditions and can affect both mechanisms of sway represented in the frequency spectrum. In future work, the parameters of acoustic/tactile manipulation should be optimized for the most effective balance stabilization, and multimodal therapies should be explored for older adults with typical age-related balance instabilities.

## Introduction

Often referred to as an inverted pendulum, upright standing is a complex task. Nevertheless, postural control remains primarily automatic, requiring little to no attentional effort (Morasso et al. 2019), relying on the dynamic control of a system of muscles, joints, and tendons working in concert (Winter 1995; Balasubramaniam and Wing 2002). Successful control of the postural system depends on sensory feedback and prediction of somatosensory, vestibular, visual, and auditory modalities (Dozza et al. 2007). However, even with this abundance of sensory information, sway variability is sensitive to subtle feedback changes from any one of these modalities (Yeh et al. 2010). Increased availability of information from one of these sensory systems can decrease postural variability and improve balance, even within aging populations (Ross and Balasubramaniam 2015, Ross et al. 2016a, b; Priplata et al. 2003). Although multisensory feedback is essential for postural control, individuals depend differentially on a combination of somatosensory, vestibular, visual, and auditory feedback for postural stability. The reliance on each of these modalities shifts as the circumstances we exist within change (Dozza et al. 2007). For example, if the availability of visual feedback is limited, such as when our eyes are closed, partial compensation in the other modalities ensures balance maintenance (Hegeman et al. 2005; Dozza et al. 2007).

Past work has shown the benefit of added unimodal noise stimulation in the somatosensory and auditory sensory systems (Priplata et al. 2002, 2003, 2006). For example, subsensory mechanical noise chips applied to the soles of the feet reduce postural sway in healthy young adults (Priplata et al. 2002), healthy older adults, and adults with central and peripheral sensorimotor deficits (Priplata et al. 2003, 2006). Although recent work shows that there are strong stabilizing effects of auditory noise on postural sway variability (Ross and Balasubramaniam 2015, Ross et al. 2016a, b), prior work on the topic showed mixed results (Hegeman et al. 2005). However, further investigation shows that the acoustic properties of the auditory stimulus might be more influential in reducing sway than if the signal offers velocity or position information (Hegeman et al. 2005; Dozza et al. 2007), which accounts for the prior mixed results. For example, Deviterne et al. (2005) found reduced sway when participants listened to prolonged speech but not when listening to a single sustained tone. Ross et al. (2016a, b) found that postural dynamics were altered based on musical properties such as the level of sensorimotor groove. These studies support that the acoustic properties of the signal might be more influential than the sound source of the signal if the signal provides a dynamic time course that can be incorporated into the dynamics of stance through sensorimotor engagement. It is unknown how multimodal auditory-tactile noise affects balance.

In assessing sway dynamics, slower and faster components of sway are often examined separately (Ross and Balasubramaniam 2015; Ross et al. 2016a, b; Yeh et al. 2010, 2014). This is because postural sway is naturally oscillatory and is composed of two primary timescales of oscillation reflecting distinct neural processes (Yeh et al. 2010). Low-frequency sway (< 0.3 Hz) is thought to reflect feedback-based corrective processes, whereas high-frequency sway (> 0.3 Hz) is thought to reflect open-loop and exploratory processes (Yeh et al. 2014). Sensory feedback-driven and exploratory sway have been shown to have a fixed cutoff frequency of roughly 0.3 Hz (van den Heuvel et al. 2009). In a study exploring the temporal relationship between body sway and a contact surface, through a light touch of the finger, it was discovered that body sway coupled with the surface when it was moved in a rhythmic fashion. It was found that the head and body sway coupled to the moving contact surface and that the coupling was nearly in-phase to frequencies of movement 0.2 Hz and lower. However, when the contact surface movement was increased to above 0.2 Hz, there was a significant lag in the coupling. This supports that lower-frequency sway relies more on sensory feedback than higher-frequency sway when there is a cutoff of 0.2 Hz (Jeka et al. 1997).

In the current experiment, we examine mean radial sway, the standard deviation of radial sway, and the high- and low-frequencies of radial sway dynamics during silence and varying modality-specific stimulation conditions. Auditory noise was provided through headphones, tactile noise was applied along the spinal column with a SubPac wearable device, and multimodal noise was provided with simultaneous auditory and tactile noise stimulation*.* Our SubPac spinal tactile noise application is novel because previous studies assessing tactile stimulation applied tactile noise to the bottoms of the feet through wearable insoles or vibrating plates in the shoes (Priplata et al 2002, 2003, 2006). All conditions were completed with and without vision to help offer validity to the data by showing the increase in variability of postural sway with no visual input, as is typical of healthy postural sway. During eyes closed, the addition of sensory noise may help to compensate for the lack of visual input which typically leads to imbalances during upright standing. The aim of this study was to examine whether multimodal noise stimulation elicits similar responses to unimodal noise on postural sway variability while eyes are open and closed. The noise in all conditions was above noticeable threshold. We hypothesized that independent auditory and tactile stimulation would lead to similarly reduced sway variability and that the combination of auditory and tactile stimulation (multimodal noise) would lead to the strongest reduction in sway variability, regardless of whether eyes are open or closed. We predicted that these effects would occur in both low- and high-frequency sway dynamics (Yeh et al. 2010), following what is reported in unimodal stimulation studies (Ross and Balasubramaniam 2015, Ross et al. 2016a, b).

## Methods

### Participants

Twenty-two healthy young adults (mean age = 21.96 ± 3.42 years) of varying heights (65.56 ± 3.48 inches) and weights (141.76 ± 27.28 lbs.) were recruited from the University of California, Merced student population. Self-report screeners were used to exclude participants with hearing impairments, arthritis, orthopedic conditions, or neurological disorders (Ross and Balasubramaniam 2015, Ross et al. 2016a, b). No participants reported recent injuries or skeletomuscular disorders, and all could stand unassisted during the experiment. The experimental protocol was carried out in accordance with the Declaration of Helsinki, reviewed by the UC Merced IRB, and all participants gave informed and written consent prior to testing.

### Experimental protocol

Participants were instructed to stand on a force platform in a relaxed, comfortable standing position with their arms at their sides and feet shoulder width apart while wearing headphones and a SubPac M2 device (SUBPAC Inc, Toronto, Ontario, Canada). Participants were given a break every 10 trials to sit down and rest their legs. Upon the continuation of the next block of trials participants were instructed again to place their feet shoulder width apart. For vibrotactile stimulation the SubPac device translated 5–130 Hz frequencies of the sound to vibrations that stimulated the body along the spinal column. The backpack’s elastic straps were tightened for a snug fit. The Subpac was set to the highest intensity setting, which created a clear vibrotactile stimulation in the tactile and multimodal conditions and was turned off during the auditory and no stimulation conditions. Participants were instructed to keep their eyes fixated on a black crosshair stimulus posted on the wall 229 cm in front of them at approximately eye level for the eyes-open trials and to keep their head facing forward and eyes closed during eyes closed trials.

The noise and silence conditions were presented in a blocked order with the visual conditions randomized within the block. Trials lasted 20 s and were accompanied by auditory white noise (intensity of 75 dB), tactile white noise, a combination of auditory and tactile noise simultaneously, or silence (10 trials with eyes open and 10 with eyes closed of each condition). Center of Pressure (CoP) was sampled at 200 Hz with an AMTI Force and Motion platform (Optima BP400600-2000 GEN 5; AMTI Force & Motion, Watertown, MA, USA). All data were collected in a single session. The auditory and tactile noise stimuli were generated using MATLAB to be random signals with a constant spectral density. Participants were exposed to the noise stimuli through both the auditory and tactile modalities separately prior to the experiment to verify that the stimuli were not uncomfortable. No participants reported discomfort at these intensities.

### Analyses

All CoP was analyzed using custom scripts in MATLAB (MathWorks, Natick, MA, USA). The first 4 s of each trial were removed to eliminate any potential startle response the participants might have had to stimulus onset. Radial sway (RS) of the CoP was calculated for each sample (i) using the anterior–posterior (A–P; *x*) and medial–lateral (M–L; *y*) components of sway following (Lafond et al. 2004):

$${RS}_{\mathrm{i}}= \sqrt{{x}_{\mathrm{i}}^{2}+ {y}_{\mathrm{i}}^{2}}$$

Average RS was calculated for each trial and was used to assess bidirectional variability in CoP during trials (Lafond et al. 2004). There are multiple other measures of postural stability that are efficient and effective when studying postural sway, including mean velocity, median power–frequency, RMS distance and sway area (Lin et al. 2008). While RS is not a direct metric of stability, it utilizes the multidirectional variability of sway to offer a more robust understanding of the sway dynamics that may lead to stability, compared to a unidirectional metric like the standard deviation of CoP magnitude or velocity (Lafond et al. 2004). Trial outliers were determined as trials with trial averages of $\pm 2$ standard deviations from that subject’s mean within condition and were removed. We removed an average of 4% of the total trials (76 out of the total 1760 trials). No subject had more than 2 trials (out of 9) removed per condition.

The effects of noise during eyes opened and eyes closed on mean RS amplitude and RS standard deviation were modeled across conditions at the group level for each analysis metric of interest using a two × four analysis of variance (eyes open/closed and silence/auditory/tactile/combined) with repeated measures and with subjects as a between factor. Bonferroni-corrected post-hoc comparisons were used to assess how individual conditions compared to one another.

The statistical analysis was then repeated using the filtered high and low frequency RS separately to assess changes in slower and faster timescales of postural control (following the methods of Yeh et al. 2010, 2014). We used low- and high-pass Butterworth filtering routines, as in Yeh et al. 2014, to decompose sway into low (< 0.3 Hz)- and high (> 0.3 Hz) -frequency sway. The filter cutoff was chosen based on van den Heuvel et al. 2009 to separate into sensory feedback-related sway and spontaneous/exploratory sway.

Finally, detrended fluctuation analysis (DFA) was used to quantify the sway dynamics over time (Delignières et al. 2003; Collins and Luca 1994). DFA is used to study the behavior of the timeseries of CoP. This method, first introduced by Peng et al. (1994), is a scaling analysis method that provides a scaling exponent $\alpha$, which offers information concerning the correlational properties of the CoP signal. When the DFA value exists between 1 < $\alpha$  < 1.5, the postural sway is considered antipersistent. This means that the sway moves in successive steps in random directions (a semi- random walk) and does not trend toward the same direction. Antipersistent radial sway dynamics is commonly described in healthy postural sway. This analysis was completed as in (Blázquez et al. 2010) using the same parameters. See Blázquez et al. (2010) and Delignières et al. (2003) for more details on the DFA method.

## Results

**Fig. 1:**
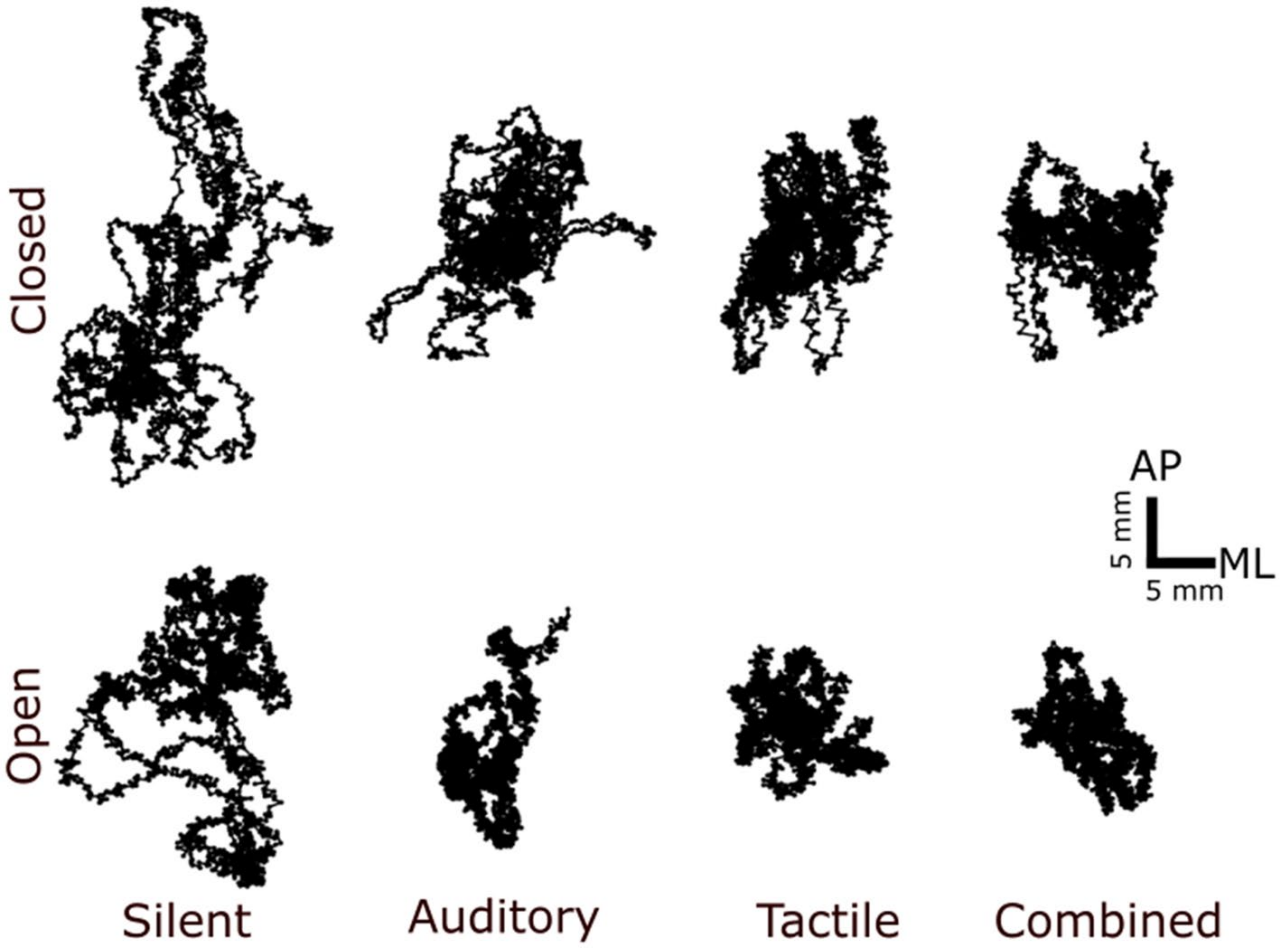
Postural sway was reduced with eyes open, with unimodal noise, and with multimodal noise in individual subjects. Center of pressure (CoP) displacement representing the effects of noise in eyes-closed/eyes-open and silent and noise conditions. With noise in both eyes-open and eyes-closed conditions, COP displacement decreased significantly

Postural sway was reduced when eyes were open and with the addition of unimodal and multimodal noise stimulation. Representative trial sway paths from each condition for the same subject are shown in Fig. 1. To demonstrate the effects of stimulation on CoP.

### Radial sway

**Fig. 2:**
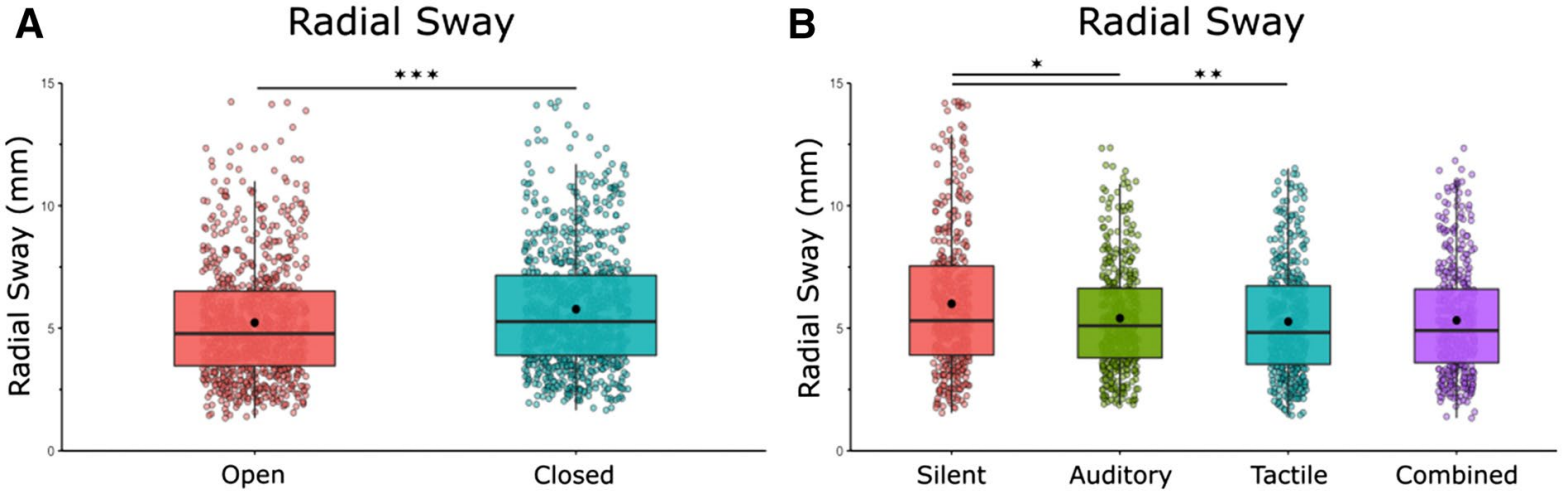
RS is significantly reduced with eyes open, with unimodal noise, and with multimodal noise at the group level. **A** Radial Sway in eyes closed/eyes open and averaged across stimulation conditions. **B** Radial Sway in silent, auditory, tactile, and combined conditions averaged across visual conditions. There was no interaction effect between vision and stimulation. Box and whiskers plot with the solid black line representing the median, the solid black dot representing the mean, and the extending lines showing the maximum and minimum values. All significant pairwise comparisons are indicated with lines between significant conditions and asterisks that mark the level of significance. (*p < 0.05, **p < 0.01, ***p < 0.001)

We found a main effect of vision [*F*(1,21) = 14.34, *η* = 0.41, *p* = 0.001] and a main effect of condition [*F*(1,68) = 6.03, *η* = 0.22, *p* = 0.001] on RS (Fig. 2). Bonferroni-corrected post-hoc comparisons were performed to compare the individual stimulation condition effects on RS when compared to silence and to other noise conditions. Post-hoc comparisons revealed a significant difference between silence (*M* = 6.00, SD = 2.88) and auditory stimulation (*M* = 5.41, SD = 2.18) *p* = 0.048, silence and tactile stimulation (*M* = 5.26, SD = 2.28) *p* = 0.005, but not between silence and multimodal stimulation (*M* = 5.32, SD = 2.27) *p* = 0.070. There was no difference between the stimulation conditions when compared to each other: auditory × tactile (*p* = 1.00), auditory × combined (*p* = 1.00), tactile × combined (*p* = 1.00). We did not find any vision × stimulation interactions [*F*(1,21) = 0.04, *η* = 0.002, *p* = 0.990].

Similarly, RS variability was reduced during all 3 noise conditions. We found a main effect of condition [*F*(1,21) = 12.58, *η* = 0.37, *p* = 0.001] on RS variability but no effect of vision on RS variability (*F*(1,21) = 0.51, *η* = 0.02, *p* = 0.484). Bonferroni-corrected post-hoc comparisons were performed to compare the individual stimulation conditions effects on RS when compared to silence and to other noise conditions. Post-hoc comparisons revealed a significant difference between silence (*M* = 2.87, SD = 0.81) and auditory stimulation (*M* = 2.18, SD = 0.58) *p* = 0.003, silence and tactile stimulation (*M* = 2.28, SD = 0.53) *p* = 0.0009, and silence and multimodal stimulation (*M* = 2.28, SD = 0.62) *p* = 0.009. There was no effect of the stimulation conditions when compared to each other: auditory × tactile (*p* = 1.00), auditory × combined (*p* = 1.00), tactile × combined (*p* = 1.00). We did not find any vision × noise interactions [*F*(1,21) = 0.09, *η* = 0.01, *p* = 0.945].

### High-frequency RS

**Fig. 3:**
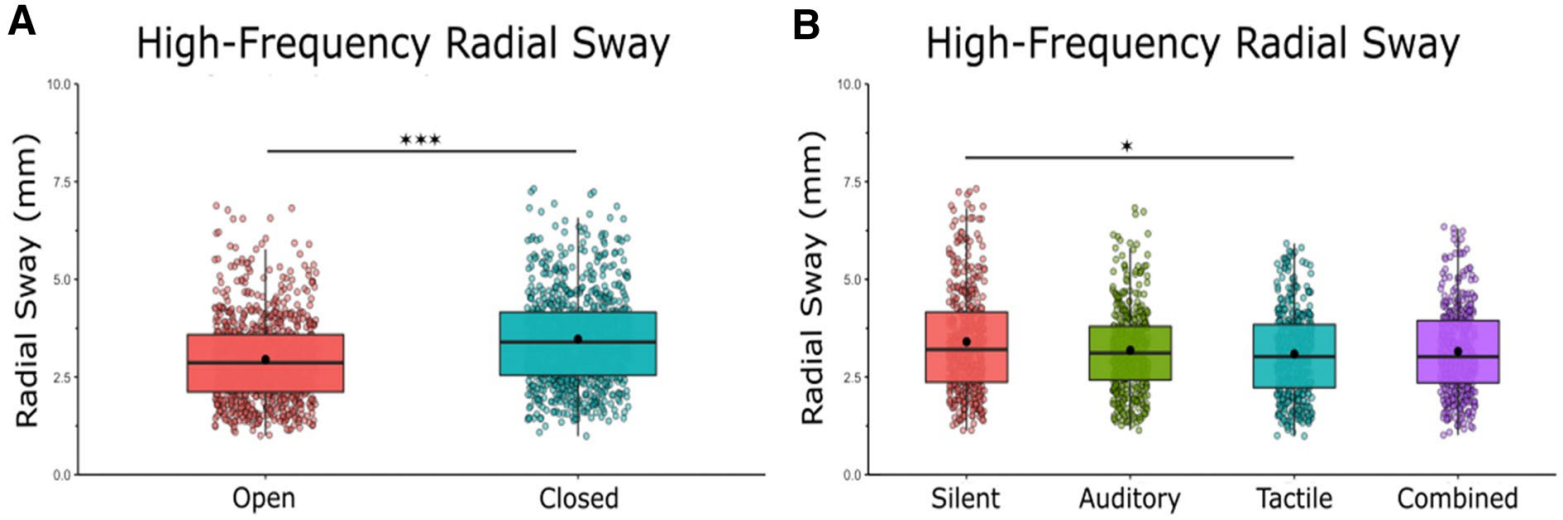
Open-loop exploratory (> 0.3 Hz) sway was reduced with eyes open, with unimodal noise, and with multimodal noise. **A** High-frequency RS in eyes closed/eyes open and averaged across stimulation conditions. **B** High-frequency RS in silent, auditory, tactile, and combined conditions averaged across visual conditions. There was no interaction effect between vision and stimulation. Box and whiskers plot with the solid black line representing the median, the solid black dot representing the mean, and the extending lines showing the maximum and minimum values. All significant pairwise comparisons are indicated with lines between significant conditions and asterisks that mark the level of significance. (*p < 0.05, **p < 0.01, ***p < 0.001)

High-frequency RS amplitude was reduced during noise conditions (Fig. 3a). We found a main effect of vision [*F*(1,21) = 42.98, *η* = 0.67, *p* = 0.001] and a main effect of condition [*F*(1,21) = 4.48, *η* = 0.18, *p* = 0.006] on high-frequency RS. Bonferroni-corrected post-hoc comparisons were performed to compare the individual stimulation conditions effects on RS when compared to silence and to other noise conditions. Post-hoc comparisons revealed a significant difference between silence (*M* = 3.41, SD = 1.32) and tactile stimulation (*M* = 3.09, SD = 1.07) *p* = 0.018, but not between silence and auditory stimulation (*M* = 3.18, SD = 1.05) *p* = 0.207, or silence and multimodal stimulation (*M* = 3.15, SD = 1.06) *p* = 0.133. There was no effect of the noise conditions when compared to each other: auditory × tactile (*p* = 1.00), auditory × combined (*p* = 1.00), tactile × combined (*p* = 1.00). We did not find any vision × noise interactions [*F*(1,21) = 0.22, *η* = 0.01, *p* = 0.879].

### Low-frequency RS

**Fig. 4:**
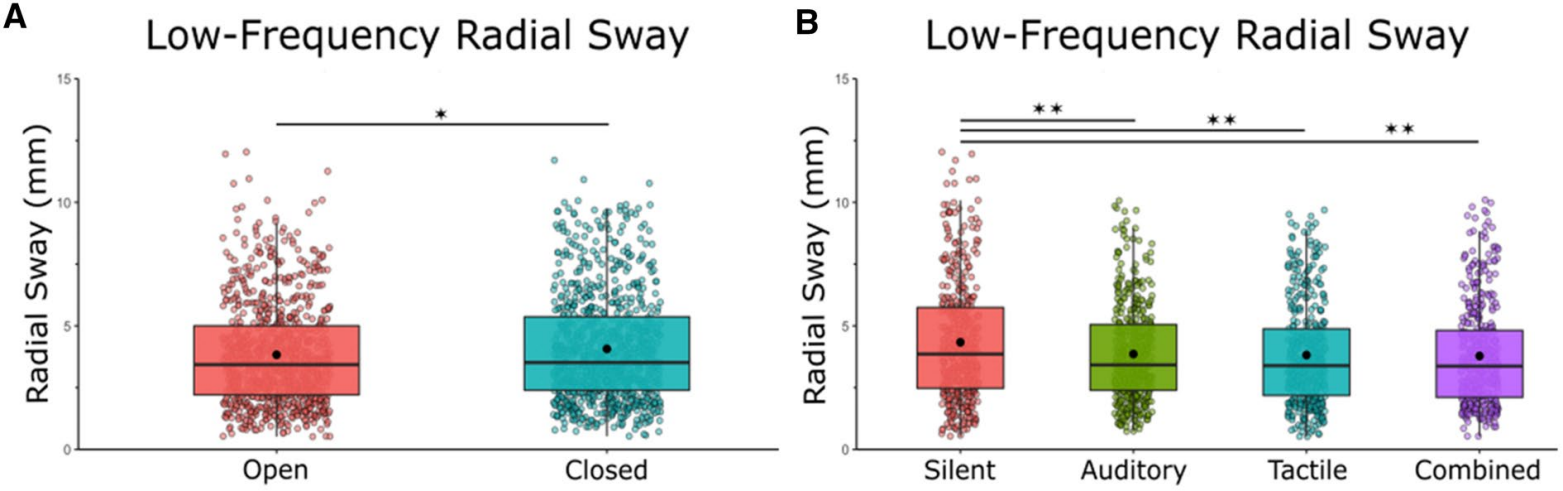
Closed-loop exploratory (< 0.3 Hz) sway was reduced with eyes open and with unimodal noise, and with multimodal noise. **A** Low-frequency RS in eyes closed/eyes open and averaged across stimulation conditions. **B** Low-frequency RS in silent, auditory, tactile, and combined conditions averaged across visual conditions. There was no interaction effect between vision and stimulation. Box and whiskers plot with the solid black line representing the median, the solid black dot representing the mean, and the extending lines showing the maximum and minimum values. All significant pairwise comparisons are indicated with lines between significant conditions and asterisks that mark the level of significance. (*p < 0.05, **p < 0.01, ***p < 0.001)

Low-frequency RS was reduced with noise in both modalities as well as in the multimodal conditions (Fig. 4b). We found a main effect of vision [*F*(1,21) = 4.64, *η* = 0.18, *p* = 0.042] and a main effect of condition [*F*(1,21) = 5.89, *η* = 0.22, *p* = 0.001] on low-frequency RS (Fig. 4a, b). Bonferroni-corrected post-hoc comparisons were performed to compare the individual stimulation conditions effects on RS when compared to silence and to other noise conditions. Post-hoc comparisons revealed a significant difference between silence (*M* = 4.34, SD = 2.45) and auditory stimulation (*M* = 3.86, SD = 2.00) *p* = 0.019, silence and tactile stimulation (*M* = 3.82, SD = 2.15) *p* = 0.017, and between silence and multimodal stimulation (*M* = 3.87, SD = 2.08) *p* = 0.019. There was no effect of the noise conditions when compared to each other: auditory × tactile (*p* = 1.00), auditory × combined (*p* = 1.00), tactile × combined (*p* = 1.00). We did not find any vision × noise interactions [*F*(1,21) = 0.029, *η* = 0.001, *p* = 0.993].

### Detrended fluctuation analysis

**Fig. 5:**
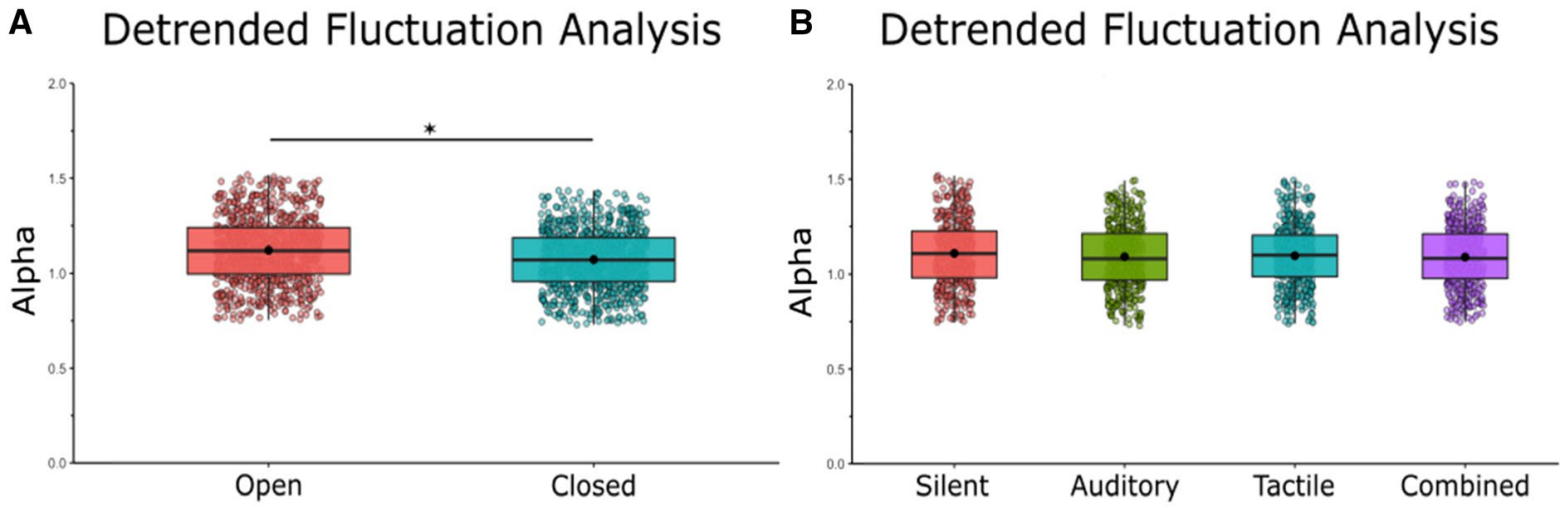
Detrended fluctuation analysis revealed an effect of vision on the random-walk pattern commonly seen in postural sway. **A** Mean *α* in eyes closed/eyes open and averaged across stimulation conditions. **B** Mean *α* in silent, auditory, tactile, and combined conditions averaged across visual conditions. Box and whiskers plot with the solid black line representing the median, the solid black dot representing the mean, and the extending lines showing the maximum and minimum values. All significant pairwise comparisons are indicated with lines between significant conditions and asterisks that mark the level of significance. (*p < 0.05, **p < 0.01, ***p < 0.001)

Detrended Fluctuation Analysis showed that RS exhibits anti-persistent fractional Brownian motion (fαm, 1 < *α* < 1.5). This semi-random walk pattern is characteristic of postural sway (Blázquez et al. 2010, Delignières et al. 2003, Collins and De Luca 1994). Within this 1–1.5 range, there are differences between subjects in *α*. We found no effect of condition on α [*F*(1,21) = 0.85, *η* = 0.35, *p* = 0.473] and a main effect of vision on *α* [*F*(1,21) = 11.55, *η* = 0.04, *p* = 0.05], indicating that with visual input, sway patterns move in successive steps in random directions (semi-random walk) and do not tend toward the same direction to a higher degree during eyes open conditions (Fig. 5A, B). We did not find any vision × noise interactions [*F*(1,21) = 0.11, *η* = 0.01, *p* = 0.952].

## Discussion

We show a reduction in postural sway amplitude and variability with auditory, tactile, and multimodal noise in healthy young adults when compared to silence, and a change in sway dynamics between eyes open and eyes closed conditions. We find no significant differences in sway amplitude or variability between the three noise conditions. These results support that postural sway variability is decreased when eyes are open and with the addition of sensory noise regardless of the modality of noise input. Vision influenced the complexity of postural sway dynamics, but the sensory noise conditions did not disrupt the typical random-walk pattern of postural sway. A large body of literature on postural sway shows that sensory information is integrated into balance maintenance in real time (Balasubramaniam and Wing 2002; Dozza et al. 2007; Wing et al. 2011), and that sensory feedback delays effect the low and high frequency components of sway differently (Yeh et al. 2010; van den Heuvel et al. 2009). Slower timescales of sway are thought to reflect drift of the inertial mass of the body (Winter et al. 1998) and are more susceptible to changes in sensory feedback (Yeh et al. 2010, 2014; van den Heuvel et al. 2009). Faster timescales of sway are interpreted as smaller adjustments around the center of mass that are more directly related to joint rigidity and muscle activation (Kiemel et al. 2005; Peterka 2002). By separating the low- and high-frequencies of postural sway, the two timescales of sway can be examined more thoroughly (Yeh et al. 2010, 2014; van den Heuvel et al. 2009). Our results show that vision and auditory noise stimuli can influence both timescales of sway regardless of the modality in which the stimulation is presented. Our data support the notion that sensory noise can reduce sway variability and this effect is present regardless of modality.

The combination of auditory and tactile noise was predicted to decrease sway variability more than auditory or tactile noise alone, but this hypothesis was not supported by our data. The explanation behind this finding is unclear, but the implications are important. Noise interventions for improving balance may be relevant and effective regardless of the modality of presentation, which would make interventions more accessible for patients with sensory impairments. If auditory noise is not possible, like in the case of hearing loss, tactile noise may be used instead. If somatosensory deficits limit using tactile noise, auditory noise may be used. In the case of both auditory and somatosensory impairments, a multimodal approach may prove to be most effective. These hypotheses need to be tested in the relevant populations.

SR is one possible explanation for the noise effect on postural sway that appropriately fits these data and previously reported results. The theory of SR explains the amplification of information-carrying signals through the addition of broad-spectrum uncorrelated noise in a threshold-based system, such as and including the nervous system (Hanggi 2002). A commonly held view of noise is that it obscures signals and needs to be filtered out to increase the signal to noise ratio. However, evidence shows that noise can contribute to signal optimization in threshold-based systems. This idea was first theorized by Benzi et al. (1981) when attempting to model the periodicity of the earth freezing and reheating by utilizing an accumulation of noise in the form of daily temperature shifts. SR has since been explained in general theoretical terms requiring 3 main phenomena: (1) a weak information-carrying signal, (2) a threshold-based system in which a barrier must be reached for information transfer, and (3) background noise (Hänggi 2002).

Subsequent work looked into the application of this theory on biological models. Research into SR within the biological systems started with benchmark publications in the early 1990s wherein the SR phenomenon was revealed in sensory neurons that were subjected to external noise (Longtin et al. 1991; Bulsara et al. 1991; Chialvo and Apkarian 1993). Certain sensory neurons are ideally suited to exhibit SR phenomena as they are intrinsically noisy and operate through threshold-based systems. In these neuronal systems, a propagating action potential breaches the necessary threshold thus triggering a firing spike, followed by a time interval which no firing occurs. This work brought SR to the attention of a much wider community and led to the application of SR on animal models. Russell et al. (1999) reported the role of noise for functional behavior with experiments on the feeding behavior of paddlefish by placing paddle electrodes on the paddlefish upon which random electrical noise was applied. The researchers assessed the spatial distribution of strike locations where paddlefish caught plankton. Upon varying the level of the noise stimulation, the authors found that the distribution began to widen, reach a maximal width at an optimal noise dose, and subsequently narrowed again with still increasing noise amplitude. Work in SR shows how nervous systems in several species utilize noise to optimize perception (Collins et al. 1996; Hidaka et al. 2000; Douglas et al. 1993; Levin and Miller 1996; Russell et al. 1999). Although the theory of SR may help to explain the behavioral changes in postural variability, it is but one possible explanation for these data.

Outside of the postural domain, work by Abedanzadeh et al. 2015 has shown the importance of sensory input on coordinated movements. Abedanzadeh et al. 2015 indicated that vision and proprioception play a dominant role in preserving the coordination patterns during bimanual movements, but audition was not as critical in its role in bimanual coordination. These results indicate the importance of vision and proprioception in bimanual coordination dynamics, and how additive noise can be beneficial for increasing the control of these movements.

Another explanation for the noise effect on postural sway is that there is an increased attentional arousal during stimulation, which could lead to improved balance. In addition, Cluff et al. (2010) showed that adding a cognitive task during standing leads to more automaticity in the balance process, which improves stability. However, it has also been shown that passively listening to a single sustained auditory tone does not affect postural sway (Deviterne et al. 2005), so we would not predict that auditory attention in our sustained noise conditions would drive a stabilizing effect in the current experiment.

It should be investigated whether there is a saturation effect with noise stimulation. With the presence of noise, we see a positive impact on balance stability. However, there may be lower and upper limits to noise intensity with regard to efficacy for balance stabilization. Peterka and Benolken (1995) showed the effect of sensory saturation using visual stimuli, and it is plausible that there is a similar effect with auditory and tactile noise stimulation. Therefore, it may be true that the sensory system utilizes noise only if presented within limits, and these limits should be investigated further. Our noise stimuli were presented at 75 dB, at a comfortable and perceivable volume/sensation for participants.

Although SR explains our and prior results, more research is required to determine the specific mechanisms driving this reduction in sway. Whether or not these effects are due to SR, attention, or some other mechanism, the findings have profound implications for improving balance in high-risk populations. One reason we may see that the multimodal condition does not have a more substantial effect than unimodal conditions is that there may be saturation effects of the noise, but this suggestion needs further study. With unimodal input in the auditory and tactile modalities, we see a strong reduction in sway. We see the same effects when we apply noise in both modalities at the same time, which could reflect a ceiling effect.

More importantly, this work shows how the modality of noise input may not be crucial in increasing stability in postural sway. Both the auditory and tactile stimulation along the spinal column reduced postural sway in similar magnitudes, as well as in the high- and low-frequencies of postural sway. This robustness may indicate a high potential for clinical application of the phenomenon for patients with a high risk of falls. The risk of fall-related injury exists for humans regardless of age (Balasubramaniam and Wing 2002), but with age, the risk of falls increases (Maki et al. 1990; Tinetti 2003). Falls lead to declines in health and independence for those who suffer injuries, especially in adults over 65 years of age (Priplata et al. 2003; Tinetti 2003). Postural sway is greater in older adults than in younger, and there may be numerous sources of this variability (Balasubramaniam and Wing 2002). Our results support that noise-based balance interventions may not require specificity to the modality of input to gain a positive impact from noise stimulation. By applying this study paradigm to older adults and clinical populations, we plan to explore further the impact that noise may have in clinical populations and the ecological validity of using noise stimuli to improve balance.

## Data Availability

Datasets generated and analyzed during the current study are available from the corresponding author upon reasonable request.
